# P106 From primary Sjögren’s disease to scleroderma renal crisis: a case of evolving overlap disease unmasked by corticosteroids

**DOI:** 10.1093/rap/rkag113.136

**Published:** 2026-11-03

**Authors:** Alexandra Godlee, Kevingulie Savino, Midhat Asif, Emma Derrett-Smith, Benjamin Fisher

**Affiliations:** University Hospitals Birmingham NHS Foundation Trust, Birmingham, United Kingdom; University Hospitals Birmingham NHS Foundation Trust, Birmingham, United Kingdom; University Hospitals Birmingham NHS Foundation Trust, Birmingham, United Kingdom; University Hospitals Birmingham NHS Foundation Trust, Birmingham, United Kingdom; University of Birmingham, Birmingham, United Kingdom; University Hospitals Birmingham NHS Foundation Trust, Birmingham, United Kingdom

## Abstract

**Introduction:**

Scleroderma renal crisis (SRC) is a rare, life-threatening condition which typically presents with abrupt-onset severe hypertension and acute kidney injury, requiring prompt recognition and treatment. Exposure to moderate- or high-dose corticosteroids is a well-recognized precipitating factor for SRC. Sjögren’s disease (SjD) may coexist with systemic sclerosis, creating diagnostic challenges when inflammatory musculoskeletal symptoms precede overt scleroderma manifestations. We report a case of SRC developing after corticosteroid therapy for polyarthritis in a patient with primary SjD and without overt signs of scleroderma.

**Case description:**

Our patient was diagnosed with primary Sjögren’s disease aged 51. She presented with a long history of arthralgia, fatigue, dry eyes and Raynaud’s phenomenon. Schirmer’s test was positive at 5 mm and 8mm of tear production from each eye. Salivary flow was 0.35 ml in 5 minutes. Immunology was positive for ANA, anti-Ro, rheumatoid factor and there was hypergammaglobulinaemia. An ultrasound of the salivary glands was in keeping with sialadenitis. Lip biopsy demonstrated florid focal lymphocytic sialadenitis. Her symptoms were controlled with pilocarpine as well as eye drops and creams.

Two years after diagnosis the arthralgia progressed to a symmetrical peripheral polyarthritis which was difficult to control with conventional DMARDs. After a reaction to rituximab she was given a course of oral steroids as a bridging therapy.

Soon after starting oral steroids she was admitted to hospital following an episode of acute confusion and was found to be hypertensive with acute kidney failure requiring haemodialysis. She underwent a renal biopsy which showed features of thrombotic microangiopathy. The case was discussed at the National atypical Haemolytic Uraemic Syndrome (aHUS) meeting where an autoimmune cause was suggested. On discussion with the local Rheumatology team, scleroderma renal crisis and catastrophic antiphospholipid syndrome were considered. ADAMS13 was borderline, whilst the antiphospholipid screen was negative. Scleroderma renal crisis was deemed the most likely diagnosis and high dose ramipril was started. On rheumatology review a small number of faint telangiectasia were noted on the hands. Over the following 6 months her renal function recovered sufficiently to stop haemodialysis however her connective tissue disease continues to evolve with a new facial rash felt likely to be autoimmune in origin.

**Discussion:**

This case highlights several interesting features. Firstly, the patient had an established diagnosis of primary Sjögren’s disease with no previous diagnosis of SSc. While overlap between SjD and SSc is recognised, Scleroderma renal crisis occurring in this setting has been reported very rarely. Our patient’s initial presentation was dominated by sicca symptoms and inflammatory arthritis, with only subtle features of systemic sclerosis, including Raynaud’s phenomenon and later identification of hand telangiectasia. This emphasises that early SSc may remain clinically occult and that overlap connective tissue diseases should be considered when new systemic manifestations develop.

Scleroderma renal crisis (SRC) is a rare but life-threatening complication of systemic sclerosis (SSc), characterised by abrupt onset of severe hypertension, acute kidney injury and, in some cases, thrombotic microangiopathy (TMA). Although its incidence has declined with improved recognition and management, SRC remains associated with significant morbidity and mortality if treatment is delayed. Early initiation of angiotensin-converting enzyme (ACE) inhibitors remains the cornerstone of management.

In this case the temporal relationship between corticosteroid therapy and the onset of SRC is clinically significant. Corticosteroid exposure is a well-established risk factor for SRC, particularly in patients with diffuse or previously unrecognised SSc. Although the precise mechanism is incompletely understood, glucocorticoids may exacerbate endothelial injury, vascular dysfunction and activation of the renin–angiotensin system, precipitating renal crisis in susceptible individuals. In this patient, oral corticosteroids prescribed as bridging therapy for inflammatory arthritis may have unmasked an evolving SSc phenotype.

This case adds to the limited literature describing SRC in patients with Sjögren’s disease and highlights the need for care when prescribing corticosteroids in patients with connective tissue diseases who have features suggestive of SSc. Recognition of subtle clinical signs may facilitate earlier diagnosis, reduce delays in treatment and improve renal outcomes.

**Key learning points:**

1. Primary Sjögren’s disease can evolve into an overlap connective tissue disease.

Although the patient fulfilled diagnostic criteria for primary Sjögren’s disease, the disease phenotype changed over time with inflammatory polyarthritis, renal crisis, and the later development of telangiectasia and an autoimmune facial rash.

Patients diagnosed with primary Sjögren’s should be monitored longitudinally for features suggesting an evolving overlap syndrome.

2. Scleroderma renal crisis (SRC) can occur despite minimal cutaneous features.

SRC should be considered even in patients without established diffuse skin thickening, particularly in those with evolving connective tissue disease.

3. Corticosteroids remain an important trigger for scleroderma renal crisis.

The renal crisis occurred shortly after commencing oral corticosteroids and highlights the need for care when prescribing in patients with evolving connective tissue disease.

4. Thrombotic microangiopathy (TMA) has a broad differential diagnosis in autoimmune disease.

5. Connective tissue diseases are dynamic rather than static diagnoses.

New manifestations appearing years after diagnosis should prompt reassessment.

Evolution from Sjögren’s disease to an overlap syndrome has important implications for prognosis and treatment.

